# Rural-to-urban migrations: acculturation experiences among university students in Botswana

**DOI:** 10.1080/02673843.2014.928782

**Published:** 2014-08-08

**Authors:** Mpho M. Pheko, Nicole Monteiro, Kagiso N. Tlhabano, Seipone B. M. Mphele

**Affiliations:** ^a^ Department of Psychology, University of Botswana, Gaborone, Botswana

**Keywords:** Botswana, acculturation, stress, students, rural-to-urban, migration

## Abstract

Many students in Botswana migrate from small rural villages and towns to the larger urban centres to attend university, and are subsequently required to adapt or acculturate to their new environments. However, the existing literature and research on acculturation experiences of students who migrate from rural-to-urban centres in Botswana is almost non-existent. The current study was therefore a qualitative exploratory investigation of the experiences of the students who migrate from rural-to-urban centres. Purposive sampling was used to recruit participants who contributed to a Talking Circle focus group. Researchers transcribed the interviews and used content analysis to uncover response themes. Findings indicated that the majority of students experienced some culture shock and a number of environmental and specific systemic stressors in their first two years of university life. Theoretical implications for understanding rural-to-urban acculturation and practical implications for university counselling approaches are also discussed.

## Introduction

As a consequence of admitting students from different backgrounds (tribes, social classes, religion, cultural ethnicities and nationalities from different countries), universities in Botswana have become increasingly diverse, modern and multicultural. This diversity requires the majority of the students, especially those who come from rural areas in Botswana, to adapt to the university culture and/or the urban lifestyle and to develop cultural competency in their social relations. The required adaptation, known as acculturation, may take the form of different strategies – assimilation, separation, integration and/or marginalisation. These adaptations have various social, health (Berry, 1997; Mui & Kang, 2006; Reynolds & Constantine, 2007) and even academic outcomes. Research has revealed that in situations where students have to adjust to social norms and customs of new and/or different cultures, they may experience problems such as culture shock (Desa, Yusooff, & Kadir, 2012; Nilsson, Butler, Shouse, & Joshi, 2008; Sandhu & Asrabadi, 1994), psychosocial difficulties and even intra and interpersonal conflicts (Lin & Yi, 1997). These stress reactions that result from having to adapt to a new host culture are referred to as acculturative stress (Berry, 2006; Berry & Kim, 1998; Lin & Yi, 1997; Mills & Henretta, 2001). For students, factors such as language barriers, academic challenges, financial problems and psychosocial and cultural differences have been revealed to have significant effects on acculturation (Pan, Wong, Chan, & Joubert, 2008). The acculturation process could therefore be described as a multidimensional phenomenon – encompassing physical, psychological, financial, spiritual, social and language dimensions (Mui & Kang, 2006). Acculturation may also occur at the group and the individual level, and it may impact the affective, behavioural, cognitive and attitudinal functioning of the individual and/or a group (Driscoll, 2011).

Several variables, including demographics, language and pre-migration attitudes, have been found to influence the acculturation process. According to Swaidan, Marshall, and Smith (2001), research has shown younger age, male gender, higher education, recency of migration and language fluency as being positively associated with successful adaptation. Moreover, of the four acculturation strategies, assimilation and integration have been associated with lower levels of social difficulty as compared to separation and marginalisation (Ward & Kennedy, 1994).

Studies regarding acculturative stress indicate a strong relationship between the physical and mental health status of individuals or groups undergoing acculturation. For example, in one study, Hovey and King (1996 *)* revealed that individuals with heightened levels of acculturative stress were also at risk of experiencing critical levels of depression and suicidal ideation, while others revealed a link between acculturative stress and depression (Mui & Kang, 2006; Rahman & Rollock, 2004). Furthermore, acculturative stress has been linked to higher levels of anxiety and depression symptoms among university students (Crockett et al., 2007), as well as negative impact on career aspirations and expectations (Reynolds & Constantine, 2007).

Investigations into factors that help mitigate acculturative stress have highlighted the significance of individual coping strategies and social support, particularly among younger migrants (Lee & Padilla, in press). Crockett et al.'s (2007) study revealed that both parental and peer support may buffer the impact of acculturative stress in the university student population. Active coping has been associated with better acculturation adjustment and avoidant coping associated with more symptoms of poor acculturation adjustment among university students (Crockett et al., 2007). Research has also revealed that individuals who experience lower levels of acculturative stress may actually be better able to balance the multiple identities (Lee & Padilla, in press) that become salient during the acculturation process.

There have also been reports that when experiencing problems, only a relatively small number of students seek psychological help (Wade, Post, Cornish, Vogel, & Tucker, 2011). This may have a bearing on clinically significant depression noted in individuals experiencing elevated acculturative stress. In Botswana, factors such as stigma and negative attitudes towards seeking help have been established as barriers to help seeking behaviour among university students (Pheko, Chilisa, Balogun, & Kgathi, 2013). This has significant implications for the degree of acculturative stress that may be found among Botswana university students.

To provide mental services to underserved groups, Vogel, Wester, Larson, and Wade (2006) highlight the importance of being culturally sensitive and being cognizant of variations in subscription to different demographic variables. Such considerations are important for settings such as Botswana, where some scholars have identified the need to further develop the psychology profession and to ensure that the voice and experiences of Africans are documented in the psychological literature (Moll, 2002; Mpofu, 2002; Pheko, Monteiro, Kote, & Balogun, 2013). To provide mental health services to underserved groups, researchers need to establish challenges that such diverse groups may experience, and how such groups conceptualise and deal with the identified problems.

## The University of Botswana context

The University of Botswana is located in Gaborone, the capital city of Botswana. Both the university and the capital city can be described as metropolitan and multicultural compared to the rest of the country. The University is the largest in the country and has a student and staff population of more than 21,000 – comprised of approximately 17,000 undergraduate students and 1000 graduate students (University of Botswana, 2014). A large number of the students admitted come from rural villages, making it important for the university to understand how such transitions may impact the students.

Most acculturative stress research has primarily focused on international students who in different universities across the world have had to adapt to the different cultures of host nations and universities. As such, there appears to be a gap in research regarding the intra-national acculturative stress – which is associated with adapting from rural-to-urban areas in countries such as Botswana – where rural and urban populations have distinct characteristics. Looking at acculturation as the changes that groups and individuals undergo when they come into contact with a different culture (Williams & Berry, 1991), students from rural villages in Botswana may have to adapt to the norms and realities of the city life as well as to the diverse culture at the universities.

## Aims of the current paper

To address this perceived research gap, the aim of the current study was to explore the intra-national acculturation experiences (including mental health and sociocultural aspects) of university students in Botswana. The study specifically investigated challenges that individual students potentially face when they have to urbanise in the face of a different set of cultural norms and values. Research focusing on different types of stressors among university students is critical as there are reports that college students are a high-risk population for mental health illness (Ryan, Shochet, & Stallman, 2010), particularly because the university environment may present threats to the student's emotional well-being (Wittenberg, 2001).

## Methods

The study was qualitative, exploratory and descriptive, and it employed a combination of a focus group format and the culturally embedded concept of Talking Circle. As a research method approach, a Talking Circle is an intra-group communication method that has been traditionally used by many indigenous cultures, especially the indigenous Native American communities to share information and facilitate group discussions (Chilisa, 2012; Hodge, Fredericks, & Rodriguez, 1996; Strickland, 1999; Struthers, Hodge, Geishirt-Cantrell, & De Cora, 2003). While the challenges and successes regarding the use of focus groups have been documented (Chilisa, Dube-Shomanah, Tsheko, & Mazile, 2005), it was deemed appropriate to use a format that will encourage multiple research participants to simultaneously produce data (Chilisa & Preece, 2005). It was also conceptualised that such an approach could also encourage the naturally reserved students to open up and share their experiences.

## Participants

A purposive sampling technique was used to recruit participants for this study and a total sample of *N* = 17 (12 females and 5 males) University of Botswana students participated in the Talking Circle focus group. Among the participants, there were four 4th year students, eight 3rd year students, four 2nd year students and one 1st year student. Ten of the participants grew up in rural areas, two grew up in towns and one in a city other than Gaborone, while three grew up in Gaborone city. One participant did not state their place of upbringing.

## Procedure

Participants were welcomed to the group, and the Talking Circle was introduced as a discussion group that fosters a safe, respectful non-judgemental environment for discussions by allowing each person the opportunity to speak without interruptions. The facilitators detailed research objectives, informed the participants of their right to withdraw from the research at any time and requested the participants' consent. The Talking Circle focus group participants decided that it would be better if one participant started by sharing his/her experience followed by others in a round robin technique. It was further agreed that the participants would take part in the discussion only if they were comfortable doing so; otherwise, they were to allow the next participant in the circle to carry on with the discussion.

To open the discussions, research facilitators asked the participants to share and discuss their experiences of transitioning from senior secondary school (equivalent to high school in the USA) life to university life. Participants were asked to first indicate where they attended senior secondary school (whether in a village, town or city), and further asked to reflect on their very first experiences at the university by discussing any challenges encountered, and/or opportunities. Furthermore, participants were asked to reflect on the year (out of their years in university) in which they felt that they were coping with university life. The facilitators recorded the field notes, and identified key reactions and non-verbal communication of the participants.

## Data analysis approach

To arrive at the themes discussed under findings, interviews were first transcribed verbatim by three bilingual researchers. This approach was important because it has been suggested that to ensure rigour and to establish reliability, more than one researcher should analyse data collected from focus groups (McDaniel, 1996). The process was also done following the interpretative phenomenological analysis guidelines for data analysis (Biggerstaff & Thompson, 2008) and the content analysis approach described by Krueger and Casey (2000). This entailed organising and preparing data by first transcribing it onto Microsoft word and then using a table to organise and compile all the responses. The second step entailed reading all the data and identifying related and recurring responses followed by a step where all the related responses were grouped and merged into themes.

## Findings

The dominating themes that emerged from the testimonies, and statements, made revealed that the majority of students – especially those who relocated from the rural villages – experienced some culture shock in their first two years of university life. Themes comprised of the following: perception of rudeness, reactions to dressing styles, reactions to greetings and salutations, experiences of stress associated with separation from family, challenges with using public transport and the use of English language.

## Perception of rudeness

Participants from the villages indicated the awkwardness that came with witnessing city students not speaking politely to their Lecturers (in this context, the title Lecturer is used to refer to the university Lecturers, Instructors and Professors). These participants pointed out that to them, Lecturers are elders and they were taught to always respect elders. Such experiences are highlighted in the following quotes:

What I also found awkward was how the students communicated with their Lecturers; I still find it hard to be rude to a Lecturer …. (Female, 4th year)

I also found students here to be rude to Lecturers. I was taught to always respect my elders …. (Female, 4th year)

## Dressing style

Another theme that emerged from participants who mainly grew up in the village was that of dressing style. They indicated the difficulty of coming to terms with the way city dwellers particularly women dress. Participants stated the following:

There are specific acceptable dress modes. In Gaborone, women wear clothes that are too short and some show private parts like cleavages which still make me uncomfortable …. (Female, 4th year)

The dress mode at UB was difficult to cope with. I grew up in a Christian community where people dressed conservatively …. (Female, 3rd year)

## Salutations

In harmony, participants who grew up in villages and towns also echoed the lack of greetings as a challenge they faced. The culture of not greeting others in the city was indicated to be quite a contrast to what happens in villages. Participants pointed out that it was not just about failure to greet others; rather, it was also about how city dwellers responded to or looked at the person who greeted them. This culture shock is depicted in the following quotes:

In Gaborone people do not greet each other, and when you greet strangers they look at you funny, so I had to cope. (Male, 4th year)

I come from a small Christian village where everyone greets each other nicely and lovingly. (Female, 4th year)

## Separation from family

For many young people who are admitted to the university, it means relocating and thereby separating from the family. Many of these young people had not lived apart from their families before; hence, relocating to attend university often means separating from one's family for the very first time. Participants noted that living away from family was not easy, as it means not having the family's daily support. The daily support could be in such activities as managing the youngster's finances, monitoring their studies and helping them to manage their time. The following quotes speak to this challenge.

Time management was a bit challenging since no one told me to wake up and get ready for classes … So it was difficult to attend classes without supervision from parents. (Male, 3rd year)

The parents expected me to have matured immediately but it was difficult for me to manage things like time and finances. For the first couple of months I always ran short of cash mid-month. (Male, 2nd year)

## Using public transport

Most students especially those that come from villages indicated that prior to coming to the university, they lived in places where they walked almost everywhere. To highlight the stresses related to using public transportation, one student stated:

I am from a small village with no electricity. So when I first came here, I was terrified of almost everything. …I will always remember my first time in a combi (the most common public mode of transportation in Gaborone), I sat inside the combi for many hours because I was terrified to ask the driver to stop, thinking that people will think I was backwards. (Female, 3rd year)

## English proficiency

Participants also noted challenges relating to the use of English language. This was particularly noted by participants who grew up in the villages. Although English is an official language and a medium of communication in schools, many participants still found it a challenge, more so when they discover that other students were more fluent and proficient in their use of English. For example, one student highlighted this challenge in the following quote:

There are many students who speak nice English; so the thought of opening my mouth with my broken English terrifies me. I literally feel my body tremble and my mouth getting dry when I am asked to speak. So for me, it has been better to just keep quiet in class even though I know my participation grade will suffer. (Female, 4th year)

## Discussions

The findings of the current study suggest that acculturative stress resulting from rural-to-urban migrations does exist within the university student population and further highlights the difficulties that students are likely to encounter while attempting to adapt to the university and city lives. Some of the challenges experienced by students emanated from their past and upbringing (religious and cultural backgrounds), their environments (adjusting to both the university and the city life), and availability or lack of social support structures (parents, family and friends). Other stressors were more systemic, and were a result of the nature of processes and procedures used within the university settings such as registering for classes and adjusting to teaching instructions, approaches and formats. Similar to the Desa et al.'s (2012) study, activities such as finding a place to live, learning where and how to use public transport, deciding on where and what to shop, and registering for classes were some of the stressors that students reported.

Worthy of highlighting is that while such culture shock experiences are considered normal human responses to alien cultural environments, they can prove to be disabling to some individuals (Mumford, 1998). The impact of culture shock may even be worse in the student population as research suggests that university students are a high-risk population for mental health problems (Ryan et al., 2010; Wittenberg, 2001).

## Language

Our findings also revealed that English proficiency contributed to the students' experiences of acculturative stress. The same findings have also been reported by Poyrazli, Kavanaugh, Baker, and Al-Timimi (2004) and Dao, Lee, and Chang (2007). Such conclusions deserve attention because studies on international students' acculturative experiences have revealed that students with lower levels of fluency in English were at a higher risk for depression than their peers (Dao et al., 2007). While the present study did not investigate international students, parallels can still be drawn to rural-to-urban migration experiences.

## Social support

Lack of social support structures also seems to be one consistent source of stress for most students who are relocated to the university for the first time. Poyrazli et al. (2004) and Hovey and Magaña (2002) revealed similar findings. This is a concern because it has also been discovered that symptoms of anxiety and depression, which are common among individuals experiencing stress, may increase if the individual does not have an effective social support system (Hovey & Magaña, 2002). Furthermore, psychological factors have been implicated in governing relationships between acculturation and mental health (Williams & Berry, 1991). Research focusing on international students suggests that students with social networks adjust better to their new living environment, have better knowledge of the host culture and also adjust better psychologically than those with fewer local social networks (Kashima & Loh, 2006).

## The classroom setting

In interpreting these findings, it is also difficult to ignore the fact that most students spend a sizeable amount of time interfacing with Lecturers. This was brought up by the participants because students face challenges regarding difficulties in participating in class discussions and correcting the Lecturers, which speak more to the classroom environments. Given such insights from students, it is conceivable that the experiences of stress may also inhibit the process of learning, and may lead to failure of courses, further exposing the students to experiencing other types of stress. Accordingly, Lecturers also need to do their share of work to ensure that the diverse students benefit equally from the information they disseminate. Lecturers could also use the current study's findings to design classroom environments that are culturally responsive and more inclusive. Our personal experiences as Lecturers do suggest that teaching approaches that incorporate small group activities provide the shy students and those who lack the confidence to express themselves in English an opportunity to communicate and interact in the classroom. These findings can thus inform teaching strategies that can enhance learning and provide students with skills that will prove useful beyond university life.

Generally, the findings support the assertion that students' diverse backgrounds provide critical lenses for interpreting their life events and situations (Whatley, Allen, & Dana, 2003). As such, the seemingly distinct dynamics surrounding the individuals, such as the cultural, political, economic, moral and health factors, could be inextricably linked (Kleinman & Becker, 1998). This means that research investigating the impact and the experience of the students' hardships, including their experiences of acculturative stress, is important for both researchers and practitioners (Arrendondo et al., 1996).

## Limitations of the current study

While the current study could be the first study to empirically investigate rural-to-urban migration acculturation and associated challenges of university students in Botswana, the findings' generalisability is somewhat limited because of the design and small sample size of the Talking Circle focus groups. In future, the use of a larger sample size of participants who grew up in the village and those who grew up in the city/town could allow for comparisons of the two groups, thereby better highlighting challenges of rural-to-urban migrations. Furthermore, the current sample is limited in terms of gender balance – restricting the researchers from pointing out gender differences regarding rural-to-urban migration experiences.

## Implications and suggestions for future research

First, from the focus group Talking Circles, the students informed that there is a university programme designed to give students the skills to be competent and effective in the university environment. They, however, further indicated that this programme is not well publicised as some students became aware of it only during the focus group discussions. This concern from students suggests the university needs to come up with ways of making the programme more attractive and visible to those students who might need it the most. The challenges further suggest that research is needed to understand the effectiveness of such programmes in addressing rural–urban migration challenges.

Second, the current study was exploratory in nature; nonetheless, the findings could still be used to assist in the development of theoretical frameworks to understand the rural-to-urban acculturation experiences within the Botswana context and in other similar contexts. Specifically, the findings of this study provide important empirical information for university wide counselling professionals working with the students populations in countries similar to Botswana. Together with suggestions that individuals high in psychological well-being may manage tensions, negative thoughts, ideas and feelings more efficiently (Bhogle & Prakash, 1995); the findings may be used to inform interventions for the students populations.

Third, while researchers such as LoSchiavo and Shatz (2009) suggest that one way to consider cultural explanations for outcomes is to conduct literature reviews that include references of international works and to also use methods that tap into broader and/or diverse populations, literature review conducted prior to the current study revealed that there is limited empirical research conducted among Botswana university students to understand the impact of such migrations. Therefore, the findings from the current study provide opportunities for investigators to conceptualise future research and come up with different approaches for investigating rural-to-urban acculturation experiences. For instance, to further advance knowledge regarding university students' experiences, researchers could investigate how the students' social, cultural and environmental experiences influence their perceptions of stress. Furthermore, the role of variables such as gender, upbringing, age, spiritual beliefs and tribal differences in dealing with life changes that may require acculturation or adaptation could also be included in both qualitative and quantitative research designs.

Another area worth investigating is the assumption that all Botswana are monocultural. Such investigations are warranted because while mental health challenges may be universal, certain mental health challenges have been identified to be more common among people with relative social disadvantages (Desjarlis, Eisenberg, Good, & Kleinman, 1995) such as people in the rural parts of countries like Botswana. Researchers need to, however, be aware of the fact that in the context of Botswana, coming or having been raised up in a rural village does not necessarily mean that the individuals are less privileged or come from a lower socio-economic status. In fact, some students who come from villages do actually have a higher socio-economic status than those who were raised in the cities. Nonetheless, Desjarlis et al.'s (1995) findings coupled with the findings from the current study can also highlight how acculturative stress and mental health challenges may present differently when looking at the psycho-cultural profiles of the rural and urban populations. These findings also support the need for multicultural counselling approaches in maintaining healthy counsellor–client relationships (Overzat, 2011) even while dealing with students/clients from the same country as they may still have diverse cultures – which may include different norms, values and beliefs.

Still related to the diversity of the culture within the same country, research also suggests that students from more traditional cultures may feel distant to the host culture especially if the gap is very big, possibly leading to more adjustment difficulties (Poyrazli et al., 2004). These assertions call for research and interventions that may measure the gaps between the student's culture and the host culture – in this context, the university and city culture. This will also ensure that interventions are tailor made for specific groups of students. This may include improving the students' intercultural competence and providing students with specific skills that equip them with the ability to interface with others whose world view may vary from their own (Torres & Rollock, 2004). Generally, the findings also offer support for the American Psychological Association's suggestions that psychologists need to use cultural lenses, recognise that every individual is influenced by the historical, ecological, sociopolitical and disciplinary contexts, and to consider cultural explanations for outcomes and interventions (American Psychological Association, 2003, 2006). We conclude by therefore urging researchers in all fields to continue examining this topic. This will ensure that policy initiatives both at university level and at country level are informed by evidence-based research highlighting the rural–urban migration experiences.
